# Identification of morphological risk factors for sacroiliac joint syndrome using in vivo computed tomography—A comparative study

**DOI:** 10.1371/journal.pone.0326152

**Published:** 2025-07-02

**Authors:** Johannes G. Dinkel, Christina Wendl, Johanna Ottner, Ekaterina Noeva, Quirin Strotzer, Christian Stroszczynski, Hans-Peter Dinkel, Marco Dollinger, Andreas Schicho

**Affiliations:** 1 Department of Radiology, University Clinic Regensburg, Regensburg, Germany; 2 Department of Neuroradiology, Medbo District Hospital Regensburg, Regensburg, Germany; 3 Faculty of Medicine, Regensburg University, Regensburg, Germany; 4 Department of Diagnostic and Interventional Radiology, Klinikum Landshut, Landshut, Germany; 5 Quartz Healthcare Germany, Regensburg, Germany; AIIMS: All India Institute of Medical Sciences, INDIA

## Abstract

**Introduction:**

Sacroiliac joint syndrome (SIJS) is an important cause of lower back pain, constituting a common source of morbidity, especially in today’s ageing population. Underlying pathophysiology is complex and likely multifactorial. Previous studies have suggested characteristic morphologies of the sacroiliac joint (SIJ) shape in pain patients.

**Aim:**

To find morphological markers for SIJS in vivo by evaluating an extensive array of, particularly anatomical, measurements of the SIJ, pelvis and associated musculature using computed tomography (CT) by comparison with non-SIJS control patients.

**Methods:**

CT scans of 754 patients suffering from SIJS and 116 age-matched control patients were analyzed evaluating anatomy and musculoskeletal degeneration. Combined and gender-grouped T-tests, Mann-Whitney-U-tests and chi-square-tests were conducted. Age correlations were tested using linear regressions.

**Results:**

Pelvis and SIJ morphology differed significantly in SIJS patients when compared to the control group. Pelves were narrower and deeper, sacra were narrower and there was less sacroiliac depth. True SIJ were deeper at S1 level and less deep at S2 and S3 levels. There was more sagittal angulation of SIJ at S1, S2 and S3 levels. Furthermore, less psoas muscle volume, higher grades of fatty degeneration of the back musculature as well as increased cutis/subcutis thickness were demonstrated in SIJS patients. Grades of SIJ degeneration were higher in all evaluated portions, although moderate in overall extent.

**Discussion and Conclusion:**

Our data suggest a number of morphological markers associated with SIJS, visible in conventional CT imaging. Further studies are needed to evaluate for causality, prognostic value and potential impact of these factors on individual treatment procedure. In a high-risk population, opportunistic analyses might enable targeted preventive measures.

## Introduction

Chronic back pain is a major source of patient morbidity in western societies, aggravated by the demographic changes leading towards a progressively ageing population. In many patients, sacroiliac joint syndrome (SIJS) is a key contributing cause of lower back pain. The sacroiliac joint (SIJ) is reported to constitute the causal source of lower back pain in up to 30% of patients [1–4]. Underlying pathophysiology remains uncertain and likely constitutes a complex, multifactorial process. Diagnosis is impeded by the relative inaccuracy of existing clinical and imaging-based tests. As a result, treatment options, such as imaging-guided sacroiliac injection, can only provide pain relief for some of the patient population and may lead to trial-and-error like cascades of treatment attempts (periradicular therapies, facet joint injection or even surgery), reducing patient satisfaction and increasing systemic healthcare costs. Individual studies have shown a considerable interindividual variety in SIJ morphology and have suggested an association between joint shape and surface area with the development of pain [5]. However, overall evidence for imaging-derived risk factors for SIJS remains limited and no additional conventional imaging-derived morphological differences have been described, especially with respect to a more extensive assessment of pelvic anatomy. Knowledge of such risk factors could facilitate the diagnosis of SIJS and, potentially, the prediction of treatment response. It could help reduce unnecessary interventions and consequently treatment costs and complications. Furthermore, higher rates of treatment success would likely enhance patient compliance and satisfaction. To understand the source of SIJ pain, an in-depth comprehension of SIJ anatomy and SIJS pathophysiology is needed. In this study, we compared the SIJ anatomy of SIJS patients with control subjects lacking SIJS symptoms using computed tomography (CT) in order to define potential anatomical risk factors for SIJS.

## Materials and methods

This study was conducted according to the declaration of Helsinki. It was approved by the local ethics committee and institutional review board. Between 01/2010 and 12/2019, 1234 SIJ injections were performed on 754 patients at our institution under written informed consent. 116 control data sets were identified using the CTs of polytraumatized patient without known trauma to the pelvis or spine. Imaging data had been obtained using the CT scanners Siemens Somatom Definition Edge, Siemens Somatom Definition Flash and Siemens Somatom Sensation 16. Measurements of an array of anatomical parameters were conducted in computed tomography scans of both groups (in SIJS patients pre-interventional), using the software Siemens Syngo Imaging, see Table 2. To evaluate joint degeneration, SIJ changes were graded based on the Kellgren-Lawrence grading scale of osteoarthritis: grade 0 = no bony alterations, no subcortical sclerosis; grade 1 = doubtful joint space narrowing, initial bony alterations, initial subcortical sclerosis; grade 2 = some joint space narrowing, small to moderate bony alterations, small to moderate subcortical sclerosis, small cysts; grade 3 = advanced joint space narrowing, definite bony alterations and/or subcortical sclerosis, cysts; grade 4 = synostosis, extensive bony alterations and sclerosis. To evaluate for fatty degeneration of the autochthonous back musculature, grading was performed based on the Goutallier classification of fatty muscle degeneration: grade 0 = normal muscle; grade 1 = initial fatty streaks; grade 2 = less than 50% fatty muscle atrophy; grade 3 = about 50% fatty muscle atrophy; grade 4 = greater than 50% fatty muscle atrophy. Data access and acquisition was conducted between December 1, 2019, and December 31, 2022. While access to individually identifying data was unavoidable during the process of data collection, all further data processing was conducted after complete pseudonymization using the software IBM SPSS (Version 26, SPSS Inc, Chicago, Illinois). Groups were compared regarding age and gender distribution. Linear regression was conducted to evaluate for a correlation between age and anatomical markers. Distribution tests were conducted graphically using histograms and q-q-diagram analysis. Normally distributed measurements (dorsal sacroiliac depth, interiliacal width, pelvic width, pelvic depth, ligamentous SIJ depth at S1 level, true SIJ angulation at S1 level, SIJ angulation at S1 and S2 level and SIJ depth at S2 level) were compared using t-tests, including Levene Testing for variance equality. Metric data without normal distribution and ordinal data (age, sacral width, true SIJ depth at S1 level, cutis/subcutis depth, SIJ depth at S3 level, SIJ angulation at S3 level, psoas fatty degeneration and atrophy, fatty degeneration of back musculature and grading of degenerative change) was compared using Mann-Whitney-U tests. Significance level was set at p ≤ 0.05.

## Results

Patient characteristics are listed in Table 1. All patients identified as either female or male. Patients with SIJS and control groups differed in terms of age and gender, with a higher mean age (61 vs. 50 years, p < 0,001) and larger proportion of female patients in the SIJS group (58.9 vs. 30.2%, p < 0,001). Linear regression analysis showed no correlation between age and any of the anatomical measurements. To account for possible gender differences, additional separate analyses were performed for female and male patients.

**Table 1:** SIJS patient characteristics. *SIJS = sacroiliac joint syndrome; BMI = Body-Mass-Index; VAS = Visual Analogue Scale, SD = standard deviation.

	SIJS patients (n = 754) (± SD)	[%]
Age in years		
Median	62 (± 13)	
Range	23 - 90	
Sex		
Female	444	[59]
Male	310	[41]
BMI		
Median	29 (± 6)	
Range	17 - 52	
Previous spine surgery	371	[49]
without biomechanical alteration	243	[66]
with biomechanical alteration	128	[35]
Previous conditions		
Rheumatological	59	[8]
Metabolic	344	[47]
Diabetes	101	[13]
Osteoporosis	28	[4]
Thyreoid	143	[20]
Other	93	[13]
Malignancy	81	[11]
Permanent Medication	586	[91]
Mean number of preparations	5,5 (± 4,0)	
Pain medication	559	[81]
One preparation	244	[35]
≥ 2 preparations	315	[46]
Psychopharmaceuticals	154	[24]
Pain		
Mean intensity (VAS)	6,7 (± 1,5)	
Mean duration prior to first intervention (months)	6,7	
SIJ Interventions	1234	
Mean number of Interventions	1,64	
Patients receiving 1 Intervention	436	[58]
Patients with ≥ 2 Interventions	318	[42]
Initial probatory injection	717	[96]
Local anaesthetic	699	[98]
Bucain	691	[99]
Prilocain	4	[1]
Other	4	[1]
Corticosteroid (Triamcinolon)	718	[96]

**Fig 1:**
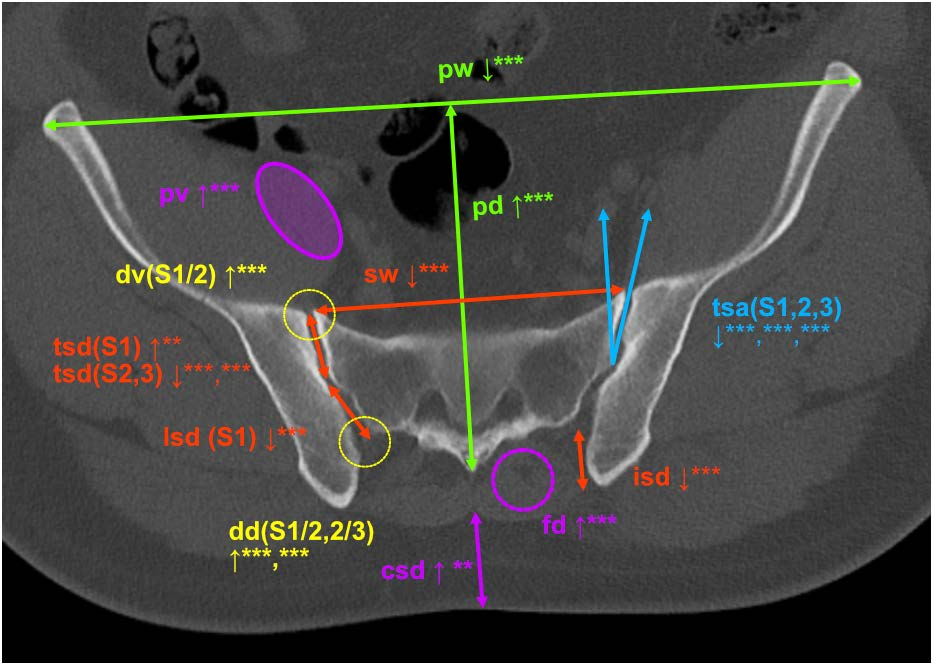
Visual overview of significant differences in anatomical parameters between SIJS patients and control group (SIJS patient, m, 35). pw = pelvic width, pd = pelvic depth, sw = sacral width, tsd (level) = true sacroiliac joint (SIJ) depth, lsd (level) = ligamentous SIJ depth, isd = dorsal sacroiliac depth (S1 level), tsa (level) = true SIJ angulation, dv (level) = degenerative changes of ventral SIJ aspect, dd (level) = degenerative changes of dorsal SIJ aspect, pv = psoas musculature volume, fd = fatty degenerative changes of the autochthonous back musculature, csd = cutaneous/subcutaneous depth. Measurements of angles in degrees (°), of lengths in mm (no further annotation). P-values summarized by asterisks: *** = p < ,001; ** = p ≤ 0,01; * = p ≤ 0,05; no asterisk annotation = insignificant p-value.

As detailed in Table 2 and summarized visually in Fig 1, the pelves of both male and female SIJS patients were on average narrower and deeper, sacra were narrower and there was less dorsal sacroiliac depth. SIJS patients exhibited a deeper true SIJ at S1 level, although differences were significant only in the male subgroup and evaluation of both genders combined. Ligamentous portion SIJ depth at S1 as well as true articulating SIJ depth at S2 and S3 was less deep in the SIJS subgroup. True SIJ angulation was lower in the SIJS group at S1, S2 and S3 levels, while there was no significant difference in angulation of the ligamentous joint portion. Less psoas muscle volume was found in the SIJS subgroup. While fatty degeneration did not differ significantly between both groups for the psoas musculature, higher grades of fatty degeneration were found in the back musculature of SIJS patients. Cutis/subcutis thickness was higher in female SIJS patients and SIJS patients overall. Higher levels of SIJ degeneration were demonstrated for SIJS patients in all evaluated localizations (S1/2 level ventrally and dorsally as well as S2/3 level), although absolute grading of degenerative change was moderate even in the SIJS subgroup. Interiliacal width did not differ significantly between SIJS and control patients.

**Table 2:** Comparison of anatomical markers and degenerative changes between SIJS and control groups for both genders.*SIJS = sacroiliac joint syndrome. dg = degrees. Values are means of individual subgroups. P-values summarized by asterisks: *** = p < ,001; ** = p ≤ 0,01; * = p ≤ 0,05; no asterisk annotation = insignificant p-value. Dorsal sacroiliac depth: distance of posterior aspect of ilium to S1, mm. Interiliacal width: posterior aspect of ilium interilial distance at S1 level, mm. Pelvic width: maximal transverse diameter, mm. Pelvic depth: sagittal diameter at midline, mm. Sacral width: ventral aspect sacral width at S1 level, mm. True SIJ depth, S1 level: a.-p. diameter of articulating joint portion, mm. Ligamentous SIJ depth, S1 level: a.-p. diameter of ligamentous joint portion, mm. SIJ depth at S2 and S3 level: a.-p. diameter of true joint in mm. True SIJ angulation, S1 level: angulation of true joint portion towards sagittal plane, degrees. Ligamentous SIJ angulation, S1 level: angulation of ligamentous joint portion towards sagittal plane, degrees. SIJ angulation at S2 and S3 level: angulation of true joint towards sagittal plane, degrees. Psoas volume: maximum axial circular area, cm ^2^. Psoas fatty degeneration: mean HU at maximum axial circular area. Back musculature fatty degeneration: degeneration of autochthonous back in grades 0-4, as described in methods section. Cutis/subcutis diameter: sagittal distance from S1 to outer cutis, mm. SIJ degeneration: degenerative changes of true SIJ at given levels in grades 0-4, as described in methods section.

	Female patients			Male patients			All patients		
	SIJS group n = 444	Control group n = 35	*Significance [p-value]*	SIJS group n = 310	Control group n = 81	*Significance [p-value]*	SIJS group n = 754	Control group n = 116	*Significance [p-value]*
Age	**61**	53	*[0,014]*	**61**	49	*** [< 0,001]	**61**	50	*** [< 0,001]
Dorsal sacroiliac depth, mm	**22,4**	26,1	*** [< 0,001]	**26,0**	29,2	*** [< 0,001]	**23,9**	28,3	*** [< 0,001]
Interiliacal width, mm	**107,7**	107,4	*[0,457]*	**97,4**	97,3	*[0,472]*	**103,5**	100,3	*[0,050]*
Pelvic width, mm	**257,6**	272,2	*** [< 0,001]	**254,1**	266,5	*** [< 0,001]	**256,2**	268,2	*** [< 0,001]
Pelvic depth, mm	**130,8**	119,6	*** [< 0,001]	**135,9**	128,7	*** [< 0,001]	**132,9**	126,0	*** [< 0,001]
Sacral width, mm	**115,5**	119,2	** [0,005]	**115,0**	117,3	* [0,020]	**115,3**	117,9	*** [< 0,001]
True SIJ depth, S1 level, mm	**20,7**	19,5	*[0,061]*	**22,3**	20,8	** [0,001]	**21,3**	20,4	** [0,006]
Ligamentous SIJ depth, S1 level, mm	**32,6**	36,5	*** [< 0,001]	**34,9**	36,9	** [0,003]	**33,6**	36,8	*** [< 0,001]
SIJ depth, S2 level, mm	**51,7**	58,4	*** [< 0,001]	**55,1**	63,1	*** [< 0,001]	**53,1**	61,6	*** [< 0,001]
SIJ depth, S3 level, dg.	**16,4**	28,0	*** [< 0,001]	**17,4**	24,5	*** [< 0,001]	**16,8**	25,6	*** [< 0,001]
True SIJ angulation, S1 level, dg.	**14,7**	18,2	* [0,03]	**14,8**	18,9	*** [< 0,001]	**14,7**	18,7	*** [< 0,001]
Ligamentous SIJ angulation, S1 level, dg.	**32,9**	32,3	*[0,842]*	**35,1**	34,0	*[0,100]*	**33,8**	33,5	*[0,551]*
SIJ angulation, S2 level, dg.	**11,0**	14,3	** [0,004]	**11,8**	15,3	*** *[< 0,001]*	**11,3**	15,0	*** [< 0,001]
SIJ angulation, S3 level, dg.	**−6,6**	−1,7	* [0,020]	**−3,0**	1,0	*** [< 0,001]	**−5,1**	0,2	*** [< 0,001]
Psoas volume, cm^2^	**5,0**	6,4	d*** [< 0,001]	**7,6**	10,7	*** [< 0,001]	**6,0**	9,4	*** [< 0,001]
Psoas fatty degeneration, HU	**55,8**	51,9	*[0,280]*	**51,6**	51,5	*[0,651]*	**54,1**	51,6	*[0,279]*
Back musculature fatty degeneration, grade	**0,53 (1)**	0,29 (0)	** [0,005]	**0,35 (0)**	0,22 (0)	** [0,036]	**0,46 (0)**	0,24 (0)	*** [< 0,001]
Cutis/subcutis diameter, mm	**36,7**	32,0	* [0,022]	**29,8**	29,5	*[0,515]*	**30,2**	33,8	** [0,003]
Ventral SIJ degeneration, S1/2 level, grade	**0,94 (1)**	0,23 (0)	*** [< 0,001]	**1,11 (1)**	0,22 (0)	*** [< 0,001]	**1,01 (1)**	0,22 (0)	*** [< 0,001]
Dorsal SIJ degeneration, S1/2 level, grade	**0,5 (0)**	0,19 (0)	** [0,006]	**0,52 (0)**	0,22 (0)	*** [< 0,001]	**0,51 (0)**	0,21 (0)	*** [< 0,001]
Dorsal SIJ change, S2/3 level, grade	**0,76 (1)**	0,4 (0)	*** [< 0,001]	**0,64 (1)**	0,25 (0)	*** [< 0,001]	**0,71 (1)**	0,3 (0)	*** [< 0,001]

## Discussion

Previous studies have demonstrated differences in the synovial surface and shape of the SIJ in pain patients and presented mathematical shape models for the identification of characteristic joint types with a possible pain associatian [5,6]. However, to the knowledge of the authors, no further potential risk factors for SIJS detectable by conventional imaging have been described in prior studies, especially in regard to a more extensive evaluation of SIJ features beyond joint surface area and shape, such as (wedge) angulation, as well as broader aspects of pelvic anatomy and associated musculature. Our data demonstrates several significant anatomical differences in the SIJ anatomy and morphology between SIJS patients and the control group.

The uneven gender distribution between the study and control group presented a potential limitation of this study, likely attributable to the higher proportion of men in polytraumatized patients. There is known sexual dimorphism in adult pelvic and SIJ anatomy, with the female pelves being smaller, wider, more uneven and less backward tilted, showing a wider sciatic notch, greater acetabular distance, smaller SIJ surface area and less sacral coverage of the articular facet of the fifth lumbar vertebra, ultimately predisposing women to SIJ hypermobility, load stress and malignment [7–9]. To avoid this gender bias, alongside a combined analysis, separate analyses were conducted for male and female patients. A potential limitation to be considered is the higher mean age of SIJS patients within the male subset and combined evaluation, a further result of the inherent epidemiological differences between SIJS and polytraumatized patients. Notably, however, linear regression did not indicate any systematic influence of age on the markers evaluated in this study. This suggests that the known age-relation of SIJS incidence, with increases in the older population, is unlikely to be sufficiently explicable by structural changes of the joint anatomy, but rather results from other factors (e.g., muscle weakness, progressive joint stress, osteoporosis or osteoarthritis). It may also indicate that relevant differences in anatomical morphology can be assessed relatively early in life. Dedicated research is needed, however, to further evaluate this inference. Importantly, this study uses conventional CT imaging, a widely available and reasonably cost-effective diagnostic tool, to identify potential risk factors. This is essential for identified risk factors to provide a meaningful impact in future diagnostic and therapeutic strategies. An inherent limitation of CT joint imaging to be considered is that it evaluates only one position, usually supine, lacking a dynamic testing component.

Musculoligamentous instability seems to be a crucial factor to SIJS development. Higher joint mobility, as well as asymmetric laxity, are known to be associated with pelvic pain [10]. An exemplification of this is the diagnostic value of Active Straight Leg Raise (ASLR) testing, a clinical assessment of pelvic stability, for pelvic pain syndrome. While healthy individuals are able to stabilize the ilium against the sacrum in a nutational position during the leg raise, SIJS patients fail to do so without a pelvic belt, resulting in counternutation [11]. Similarly, SIJS in pregnant women is tested by assessing if pelvic pain can be relieved by manual stabilization.

**Fig 2:**
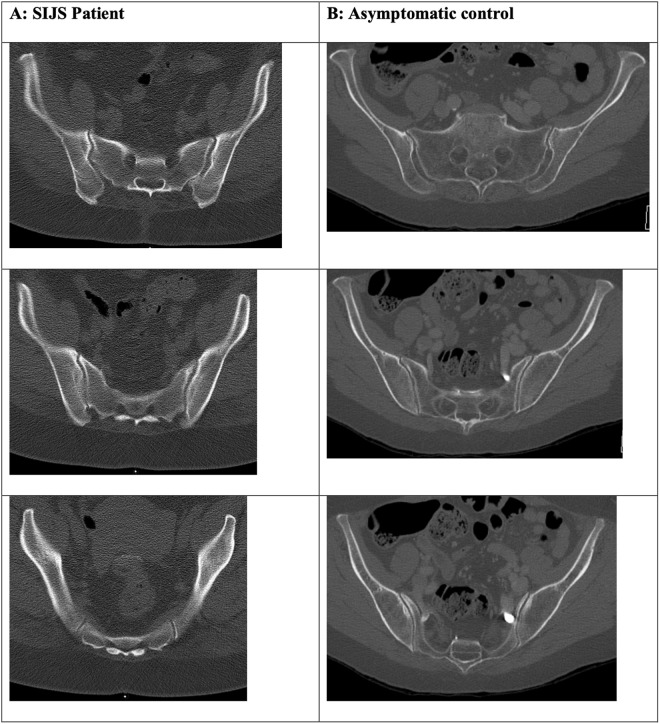
Visual anatomical comparison of “typical” SIJS patient pelvic phenotype (A; f, 52) and asymptomatic control (B; f, 67). Almost all demonstrated parameter differences of pelvic anatomy can be appreciated in this visual comparison: the SIJS patient pelvis (A) shows more sagittal angulation of the SIJ, a narrower and deeper pelvis, narrower sacrum, deeper true SIJ at S1 and less deep SIJ at S2/S3, as well as thicker subcutaneous fat tissue, lower psoas volume and a higher degree of fatty muscle degeneration.

In our study, both male and female SIJS patients had narrower and deeper pelves, narrower sacra, and reduced dorsal sacroiliac depth. It is reasonable to assume that this represents a biomechanical phenotype predisposing to stress on the sacroiliac joints and thus the development of SIJS. A narrow sacrum may alter pelvic force distribution, particularly during weight-bearing. Reduced width may indicate a smaller area of load distribution with a potential increase in focal load stress. It may furthermore provide less surface for stabilizing ligamentous attachments. Reduced dorsal sacroiliac depth is likely to biodynamically affect the SIJ alignment and articulation, potentially increasing the shift of the sacrum towards the ilia during mechanical loading, thus predisposing to joint instability or even malalignment, as well as increasing joint surface stress. It may also lead to suboptimal ligamentous attachment points impacting joint stability and function. As this metric was measured in the upper portion of the sacrum its relative reduction in SIJS patients may also be an expression of a dorsal tilt/counternutational position in the supine CT position, which is assumed to indicate joint instability and was demonstrated to be associated with SIJS in dynamic studies [12]. Characteristic phenotypes of SIJS and control patient pelvic anatomy are exemplified in Fig 2.

The SIJ shows significant variability in surface area and shape [5,13]. Its cranial portion is a true synovial joint at the anterior aspect, while the posterior aspect resembles a syndesmosis-like interosseous space stabilized by strong ligaments [14]. The auricular-shaped true SIJ allows for nutation and counternutation movements, whereas only very limited movement occurs in the ligamentous joint aspect [14]. SIJS patients in our study showed a deeper true SIJ at the S1 level compared to control patients, although differences were significant only in the male subset and combined evaluation of both genders. Ligamentous SIJ depth at S1 and true articulating SIJ depth at S2 and S3 were less deep in SIJS patients. This “cranialized” configuration of the true SIJ may allow for more freedom of movement within nutation and counternutation and may lead to uneven load distribution between the cranial and caudal joint aspects, particularly considering differences in cranial and caudal joint anatomy with a more symphysis-like construction of the lower SIJ [10,15]. Moreover, these findings are consistent with previous research: the SIJS phenotype resulting from aforementioned depth differences likely represents an indirect manifestation of the crescent-shaped “type 3” SIJ morphology demonstrated by Jesse et al. to be overrepresented among pain patients [5].

While the SIJs provide inherent stability in the lateral direction, its stability in anteroposterior direction as well as freedom of movement in nutation and counternutation are at least in part dependent on the “wedging” effect of the opposingly oblique angulation of both SIJs. Previous studies have demonstrated the influence of an increased wedge angle on SIJ stability [10]. This effect will be most pronounced in anteroposterior direction and less impactful with further deviation of the applied force vector towards the craniocaudal or lateral plane. It will also limit the range of movement in nutation and counternutation. Narrower and deeper pelves are likely a phenotype associated with less wedging of the SIJs, thus contributing to instability and, potentially, predisposing to SIJ pain. For instance, typical SIJ pain in pregnant women is believed to result from increased ligamentous laxity of the long dorsal ligament of the posterior sacroiliac ligament complex, thus providing insufficient counteraction to counternutation [16]. In our analysis, true SIJ angulation was shown to be lower in the SIJS subgroup at S1, S2, and S3 levels, suggesting a predisposition to instability due to a more sagittal orientation and, thus, reduced wedge effect between sacrum and ilia. In case of joint instability, excessive compensatory muscular activity may add further mechanical stress on the SIJ, a potential link between SIJS and general lower back pain [10].

SIJS patients also exhibited less psoas muscle volume, indicating increased muscular atrophy. While fatty degeneration did not differ significantly between both groups for the psoas musculature, higher grades of fatty degeneration were found in the autochthonous back musculature of SIJS patients. These findings align with previous studies that identified relative weakness of stabilizing musculature in SIJ dysfunction, as well as an association with impairment of the pelvic force closure mechanism [10,11,17]. While no muscles directly cause movement of the SIJ, the surrounding musculature indirectly provides important active and passive stabilization of the SIJs, especially during dynamic loading where they act as the crucial axis between the spine and lower extremities. Of note, the muscle groups evaluated in this study may be seen as representatives of a larger group of indicator muscles regarding fatty atrophy as well as regarding the SIJ stabilization (further examples including the inner pelvic musculature, hamstring and gluteal muscles) [15]. As a typical sign of muscle disuse, fatty degeneration also underscores the contribution of life style factors, i.e., lack in physical exercise, to SIJS development. This is underlined further by the higher subcutaneous fat tissue thickness demonstrated in female SIJS patients and combined analysis of SIJS patients, linking into previous studies demonstrating obesity as a risk factor for SIJS [18].

Osteoarthritic changes of the SIJ are increasingly common with higher ages and are generally regarded as non-specific for SIJ dysfunction [14,15]. Our analysis showed more pronounced joint degeneration in SIJS patients in all evaluated localizations, however with overall only moderate grades of degeneration overall. It is likely that joint degeneration is both consequence and contributor to SIJS, as patients often begin to experience relevant symptoms before the development of fulminant osteoarthritis. It should, thus, only be considered as an adjunct marker for advanced stages of SIJS.

Considering the major associated individual and socio-economic burden associated with lower back pain (i.e., work absenteeism, often prolonged history of diagnostic and interventional procedures), the identification of high-risk individuals could provide considerable benefit by enabling strategies for future disease prevention [19]. If a causal link with later development of SIJS can be confirmed in future studies, the identified morphological features may be utilized to prompt targeted preventive measures such as lifestyle changes, sports, movement and physiotherapy, whenever opportunistically available from CT scans [20]. The data and insights provided by this study, as well as previous research indicating variations in SIJ surface are and shape in pain patients, could facilitate the development of a mathematical model of the pelvi, advancing the understanding of SIJ biodynamics and the mechanisms underlying pain development [5,6]. In a clinical setting, the automated detection of such morphological markers is achievable using computer vision algorithms, providing a convenient and fast opportunistic screening tool, as demonstrated previously in applications such as osteoporosis detection [21].

## Conclusions

Our study reveals significant differences in a number of morphological markers between SIJS and control patients, most notably narrower and deeper pelves, a more sagittal angulation of SIJ, less depth of the caudal SIJ aspect and higher grades of degenerative joint change as well as fatty muscle atrophy. These findings suggest that anatomical markers identifiable on conventional CT imaging may represent risk factors for SIJS, aid in the often challenging diagnosis of SIJS as the underlying cause of pelvic or lumbar pain, and thus allow for more efficient, personalized treatment approaches. In a high-risk population, opportunistic analyses might enable targeted preventive measures. However, further studies are needed to clarify causality and assess the impact of targeted interventions for improved patient outcomes.

## Supporting information

S1 File Ethics approval. English translation of the original German Ethics Review Board approval document. (DOCX)
