# Supplementary material for: Identification of morphological risk factors for sacroiliac joint syndrome using in vivo computed tomography—A comparative study
Source: PLoS One. 2025 Jul 2;20(7):e0326152. doi: 10.1371/journal.pone.0326152 (PMC12221093; doi:10.1371/journal.pone.0326152)
Supplement: S1 File — (DOCX) [file pone.0326152.s001.docx]

**Translation of Ethics Approval**

### "Consultation in Accordance with § 15 (1) of the Professional Code for Physicians in Bavaria for the Retrospective Research Project Entitled:

**Evaluation of the Effectiveness and Adverse Effects of CT-Guided Infiltration of the Sacroiliac Joint, with Special Consideration of the Biomechanics of the Pelvic Skeleton**

**Applicant:**
PD Dr. med. Andreas Schicho

**Institution:**
University Hospital Regensburg, Institute for Radiological Diagnostics

The Ethics Committee at the University of Regensburg conducted a consultation in accordance with § 15 of the Professional Code for Physicians in Bavaria for the above-mentioned research project in its meeting on 13.11.2019, using a simplified procedure. Following a cursory factual and legal review, as well as an assessment of the benefit-risk ratio, the committee raises

**no professional-ethical or legal concerns regarding the implementation of this research project.**

The decision was based on the application form, including a brief textual project description dated 10.11.2019.

**Notes:**

1. The medical and legal responsibility for conducting this research project and for the accuracy of the information provided in this consultation process remains solely with the researcher(s) advised by the committee. The obligation to independently comply with relevant laws and regulations is explicitly noted.
2. The Ethics Committee at the University of Regensburg confirms that it operates in accordance with applicable laws, regulations, and the current version of the GCP/ICH guidelines. Individuals involved in the study did not participate in the decision-making process.
3. The decision was made using a simplified procedure because, according to the applicant, the research:
   - Does not involve the use of pharmaceuticals and/or medical devices
   - Does not involve radiation exposure
   - Does not require study-related patient or subject contact
   - Is purely retrospective and does not allow external persons access to source data
   - Does not involve data collection outside the researcher's own institution
   - Has no expected immediate consequences for specific patients based on the research results
   - Involves no clinical interventions on patients and/or subjects
   - Does not include data collection beyond the evaluation of medical records and their annexes
   - Has no external project partners
   - Is not conducted within the framework of contract research
   - Does not involve the use of biological materials
4. In the simplified procedure, the following additional conditions apply, which the applicant is hereby informed of:
   - No subsequent amendments can be processed. A new application must be submitted if changes are required.
   - No subsequent confirmations or copies of this letter can be issued. The original letter must therefore be kept carefully.
5. No fees will be charged for this procedure."
